# Dataset from PPG wireless sensor for activity monitoring

**DOI:** 10.1016/j.dib.2019.105044

**Published:** 2019-12-23

**Authors:** Giorgio Biagetti, Paolo Crippa, Laura Falaschetti, Leonardo Saraceni, Andrea Tiranti, Claudio Turchetti

**Affiliations:** Department of Information Engineering, Polytechnic University of Marche, Ancona, Italy

**Keywords:** Photoplethysmography, Accelerometer, Machine learning, Activity recognition

## Abstract

We introduce a dataset to provide insights about the photoplethysmography (PPG) signal captured from the wrist in presence of motion artifacts and the accelerometer signal, simultaneously acquired from the same wrist. The data presented were collected by the electronics research team of the Department of Information Engineering, Polytechnic University of Marche, Ancona, Italy. This article describes data recorded from 7 subjects and includes 105 PPG signals (15 for each subject) and the corresponding 105 tri-axial accelerometer signals measured with a sampling frequency of 400 Hz. These data can be reused for testing machine learning algorithms for human activity recognition.

**Table:** Specifications Table

Subject	Electrical and Electronic Engineering Biomedical Engineering
Specific subject area	Photoplethysmography (PPG)
Type of data	Data matrix, table, image
How data were acquired	PPG and accelerometer signals were acquired using the Maxim Integrated MAXREFDES100 device applied to a wrist band.
Data format	Raw mat files.
Parameters for data collection	Participants were familiarised with the experimental protocol by testing the equipment and software prior to recording.
Description of data collection	Participants performed five acquisition sessions each of squat exercises, stepper exercises, and resting. PPG and acceleration signals were concurrently recorded during the voluntary activity.
Data source location	Institution: Università Politecnica delle Marche, Department of Information Engineering, via Brecce Bianche, 12 City/Town/Region: Ancona (AN) Country: Italy Latitude and longitude (and GPS coordinates) for collected samples/data: 43°35′12.9″N 13°31′00.5″E
Data accessibility	With the article.

**Table:** 

**Value of the Data** • The data provide a collection of photoplethysmography (PPG) signals synchronized with the accelerometer signals [[1], [2], [3], [4]]. • The data are suitable for different pattern recognition and classification tasks to detect different activities (such as squat or stepper) from rest [5,6]. • The dataset is suitable to signal processing analysis of the PPG signal, in order to investigate motion artifact reduction techniques [[7], [8], [9], [10], [11]].

## 1 Data

The dataset provided with this article supplies valuable information to investigate the PPG signal acquired from the wrist by using the Maxim Integrated MAXREFDES100 device.

The dataset consists in an archive file named “PPG_ACC_dataset.zip”, containing a folder for each subject (S1, …, S7) and 30 raw mat files for each folder, for a total of 210 raw mat files corresponding to each recording session of each subject. The mat files (named “<activity><N>_acc.mat” and “<activity><N>_ppg.mat”, where <activity> = “rest”, “squat”, “step”, and <N> = “1”, … , ”5”) contain one data matrix whose first column is the sampling time [s]. The other columns represent the measure of the PPG signal or the measure of the three axes accelerometer signal. The PPG signal values correspond to the ADC output of the photodetector with a pulse width of 118 μs, a resolution of 16 bits and a full-scale range of 8192 nA, lighted with the green LED. The three axes accelerometer signal values correspond to the MEMS output with a 10-bit resolution, left-justified, ± 2g scale.

The dataset contains 210 recording sessions for a total duration of 17201 s. Table 1 shows the details about the consistency of the dataset, in terms of duration.

**Table 1:** Data consistency: Acquisition time for each subject.

Subject ID	Squat Activity [s]	Stepper Activity [s]	Resting Activity [s]
1	311.5975	442.9900	3271.7
2	216.7975	397.6150	2962.8
3	231.4950	271.0400	1323.8
4	212.5750	269.6800	1361.9
5	246.2950	241.9750	1440.9
6	237.3700	325.9025	1402.0
7	266.8600	254.9300	1510.7

**Fig. 1:**
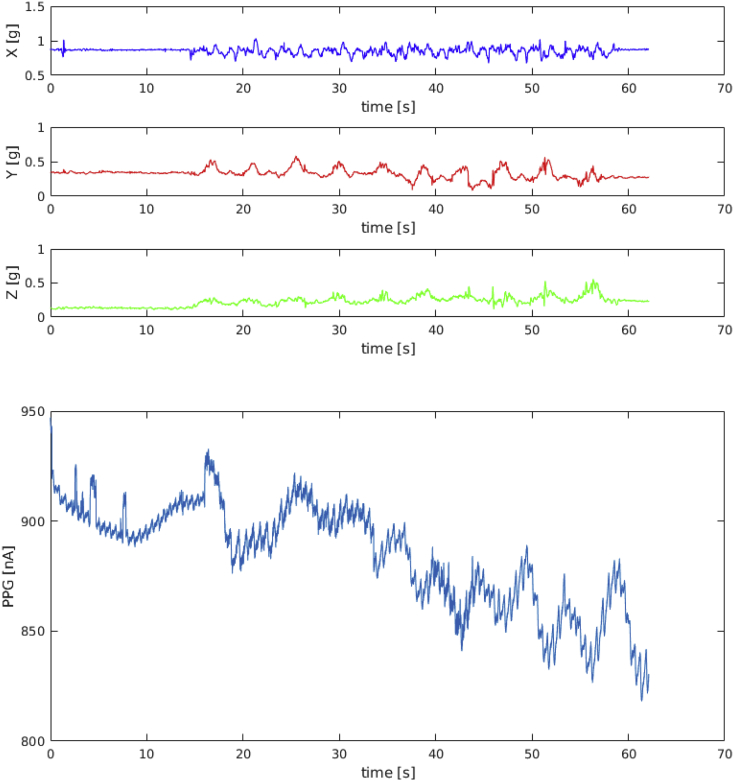
Data recorded from subject ID1 during squat activity.

**Fig. 2:**
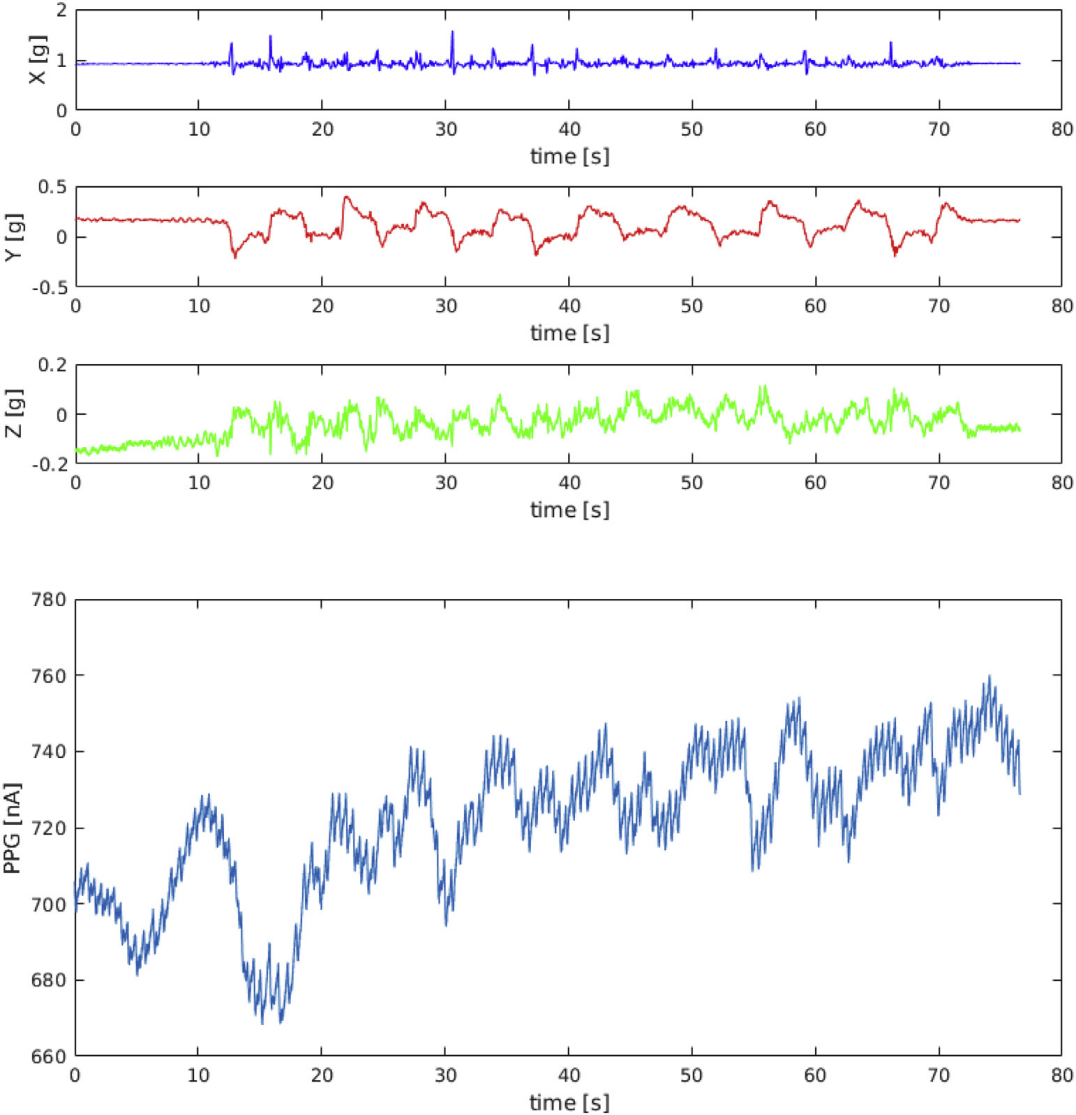
Data recorded from subject ID 1 during stepper activity.

**Fig. 3:**
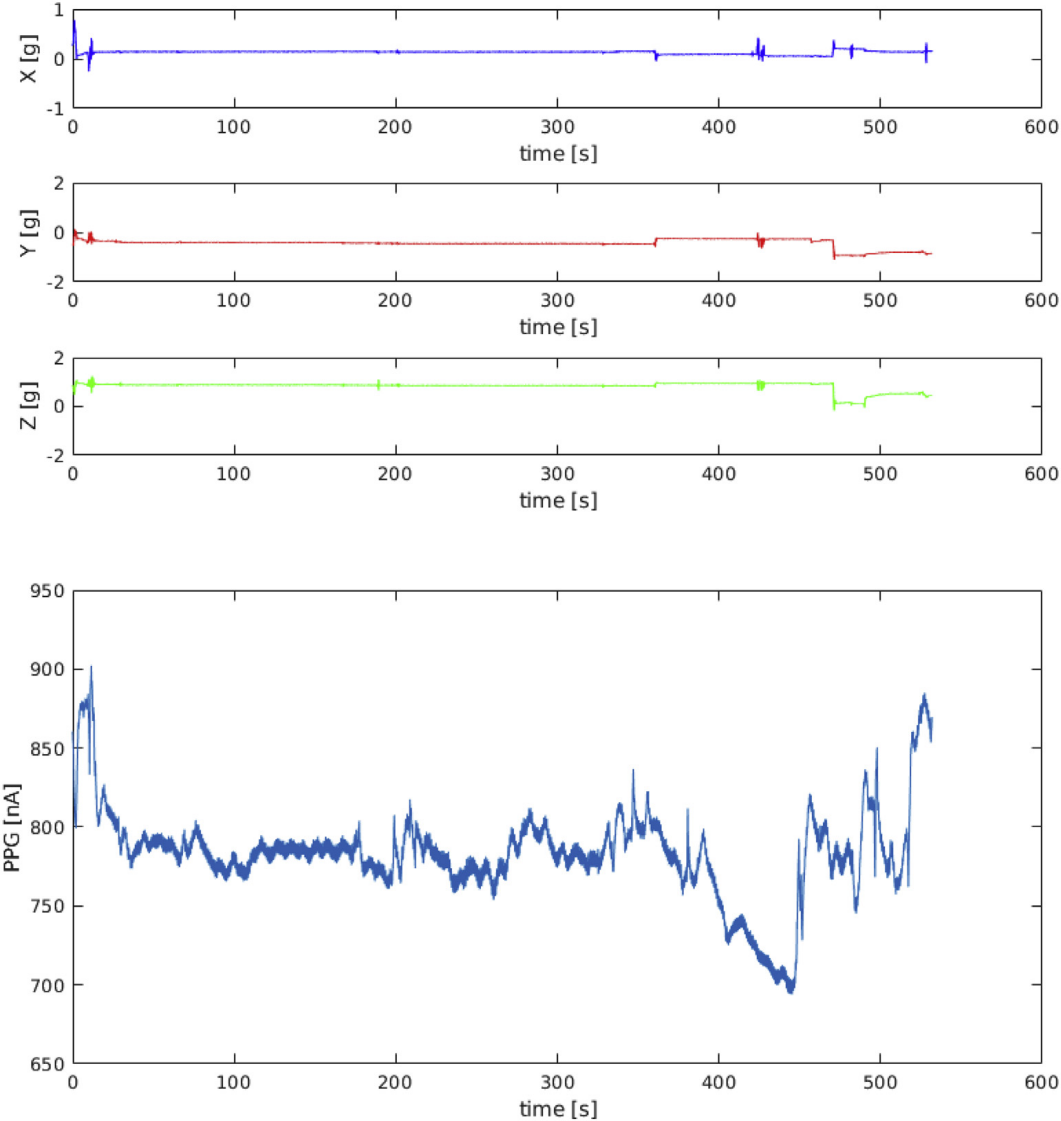
Data recorded from subject ID 1 during resting activity.

Fig. 1, Fig. 2, Fig. 3 show the tri-axis accelerometer signals and the PPG signal for subject ID 1 during a session of squat, stepper and resting activities, respectively.

**Fig. 4:**
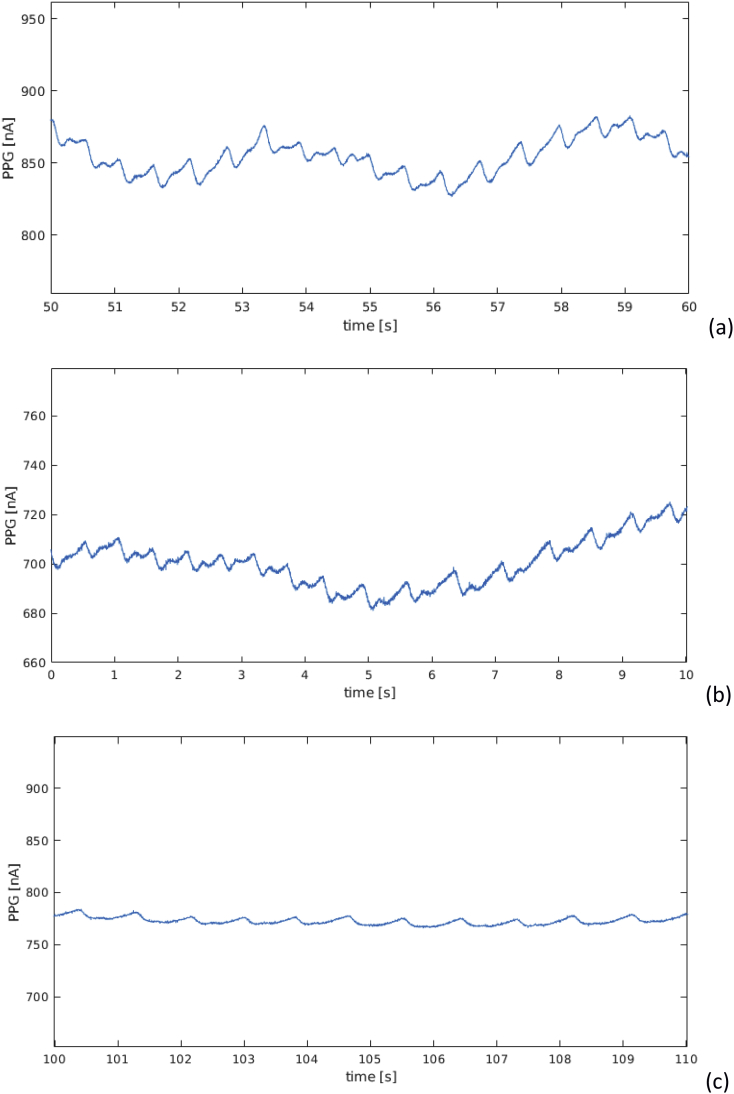
PPG signals recorded from subject ID 1 during 10 s of (a) squat, (b) stepper, and (c) resting activities.

Fig. 4 reports the same PPG signals for subject ID 1 for a window of 10 s.

## 2 Experimental design, materials, and methods

The experimental protocol used to acquire the data, for every subject can be resumed as follows: seven adult subjects volunteered to perform exercises for data acquisition.

The material has been acquired by performing the following activities:

• Five series of ten squat exercises each;
• Five series of ten stepper exercises each;
• Five series of resting for five minutes each.

### 2.1 Participants

A total of 7 subjects that includes 3 males and 4 females aged between 20 and 52 years were recruited for participation as reported in Table 2.

- Age = 31.5714 ± 13.6120 years old
- BMI = 23.5429 ± 2.5310 kg/m^2^.

**Table 2:** Subjects.

Subject ID	Height [m]	Weight [kg]	BMI [kg/m^2^]	Age	Sex
1	1.73	70	23.4	22	M
2	1.78	72	22.7	22	M
3	1.80	80	24.7	44	M
4	1.70	60	20.8	52	F
5	1.65	55	20.2	20	F
6	1.57	66	26.8	41	F
7	1.78	83	26.2	20	F

The subjects were selected from adult healthy people.

A detailed written consent was obtained from all participants.

### 2.2 Procedure

The PPG signals were recorded during the voluntary activity from the wrist by using the Maxim Integrated MAXREFDES100 device.

**Fig. 5:**
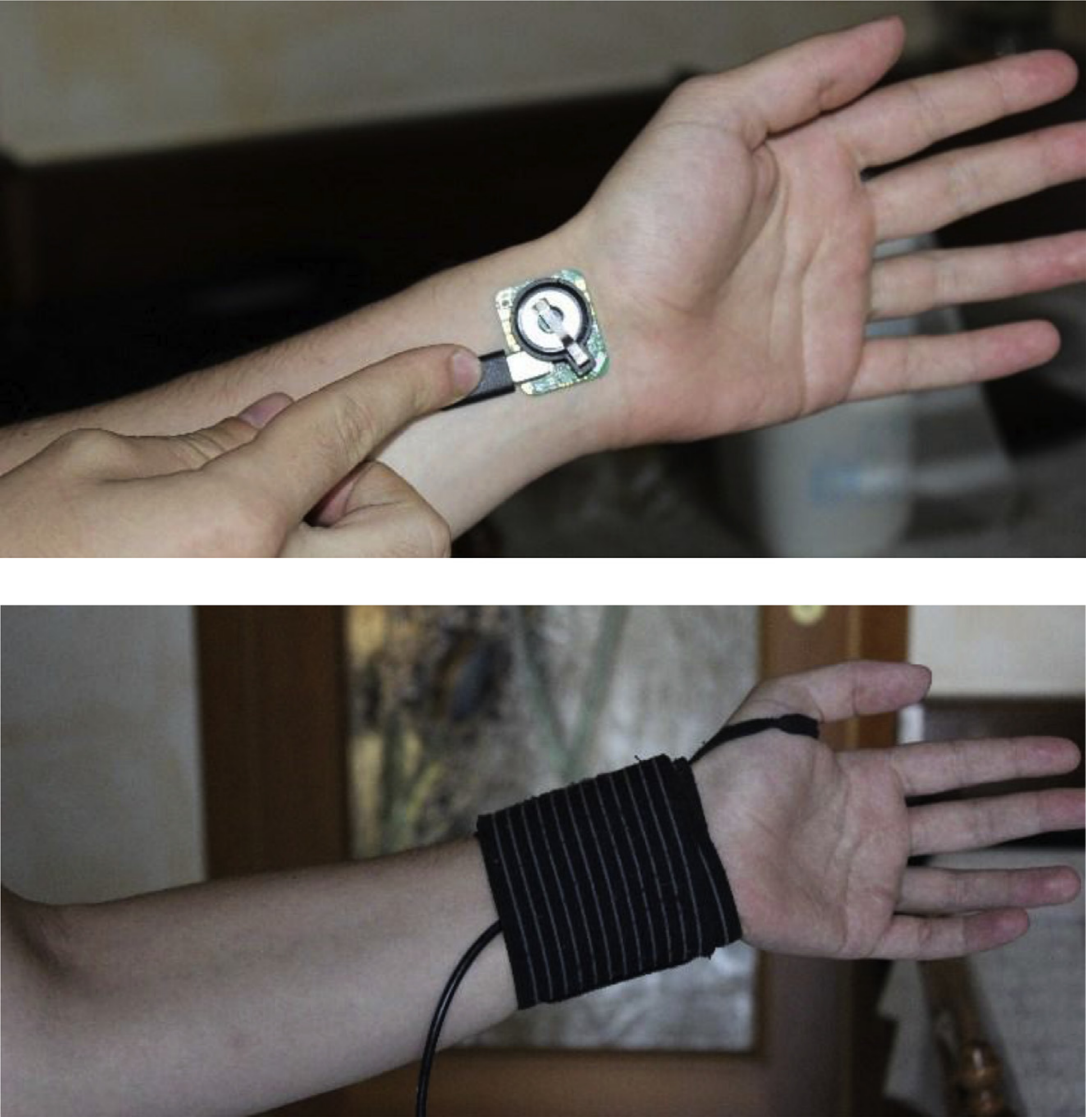
PPG sensor placement.

For applying the device directly on the wrist, a specific weight lifting cuff has been used (see Fig. 5 as a reference), adjustable by a tear-off closure, with excellent elastic properties that make it particularly suitable to guarantee a perfect adherence of the sensor device to the skin surface. The sensor was then initially fixed on the wrist of the subject, to then be fixed by adequately tightening the band, with the cable (used in the "tethered" mode) that comes out from the rear end of the band.

Particular care has been devoted to all the phases of preparation of the measurement set-up: *i)* the correct positioning of the sensor inside the sports belt, *ii)* the correct wiring, checking that it is securely connected inside of the default socket, and that it is also well locked in the support, so as to ensure that it does not move when performing various types of activities. Loss of adherence to the skin-device interface would cause the addition of high-frequency noise in the acquired signals, making them unusable.

The signals were acquired with a sampling frequency of 400 Hz.
